# A Novel *Mycobacterium cosmeticum*-Like Bacterium Isolated from the Ear Swab of a Patient with Otitis Externa

**DOI:** 10.1155/2015/825819

**Published:** 2015-03-17

**Authors:** Jeanette W. P. Teo, Janet W. S. Cheng, Roland Jureen, Raymond T. P. Lin

**Affiliations:** Department of Laboratory Medicine, Microbiology Unit, National University Hospital, Singapore 119074

## Abstract

We describe the identification and characterization of a novel nontuberculous mycobacterium (NTM), isolated from an ear swab of an adult male patient with chronic otitis externa. Genetically, the bacterium is most closely related to *Mycobacterium cosmeticum*; however, growth and biochemical features indicate that it is distinctly different. Here, we highlight for the first time an unusual NTM that is a probable cause of ear infection.

## 1. Introduction

Nontuberculous mycobacteria (NTM) are known albeit uncommon causes of otic infections (otitis media and otomastoiditis). Members of the* Mycobacterium abscessus* complex are typical causative agents [1, 2], although on rare occasions* M. avium* [3] and* M. fortuitum* [4] have also been isolated from patients with disease. Likewise, otitis externa can have a bacteriological component associated with the condition, with* Pseudomonas aeruginosa* and* Staphylococcus aureus* being most commonly implicated [5, 6]; however, the condition has never been attributed to* Mycobacterium* spp.

## 2. Case Report

In this report, we describe the isolation of a novel* M. cosmeticum*-like bacterium (NTM165). The isolate was obtained from an ear swab of a 39-year-old male patient with chronic otitis externa. No other microorganism grew from the specimen and NTM165 was diagnosed to be clinically significant. As this was a referral sample from a local polyclinic, we were unable to obtain further clinical details with regard to history and treatment. Ziehl-Neelsen staining indicated that the bacilli were acid-fast. After 3 days of growth at 37°C on Middlebrook 7H10 agar plates, NTM165 colonies were smooth and off-white color. No growth was observed at 42°C. Matrix-assisted laser desorption ionization-time of flight mass spectrometry (MALDI-ToF MS) identification of NTM165, with full protein extraction procedures, was performed as described by the manufacturer (Bruker Daltonics, Bremen, Germany). A result score of 1.727 matching* Mycobacterium cosmeticum* was generated; however, this was an inadequate score to confidently define the species. The isolate was sent to a local reference tuberculosis laboratory for further identification by high-performance liquid chromatography (HPLC) analysis. Evaluation of the mycolic acid pattern by HPLC indicated that NTM165 had a profile similar to the* M. fortuitum* complex but provided no further identification. Mycobacterial commercial DNA probe assays, GenoType Mycobacterium CM/AS (Hain Lifescience GmbH, Nehren, Germany) and INNO-LiPA MYCOBACTERIA v2 (INNOGENETICS N.V., Gent, Belgium), yielded no identification for the NTM.

For molecular identification, the mycobacterial DNA was extracted according to the DNeasy Blood and Tissue Kit (QIAGEN, Hilden, Germany) protocol for Gram-positive bacteria. The full length 16S rRNA, 16S-23S internal transcribed spacer (ITS), and partial* rpoB* gene and* hsp65* genes [7, 8] were amplified and sequenced. The gene sequences were compared to those available in the GenBank database using BLASTN and an approximate phylogenetic affiliation was determined for NTM165. Phylogenetic trees were built using MegAlign software (DNASTAR, Madison, WI) and analyzed by bootstrap analysis with 1000 resamplings and 111 seeds. The full 16S rRNA gene sequence of NTM165 showed highest level of sequence similarity (1440/1456 nt, 99%) to* M. cosmeticum* type strain LTA-388 (NCBI Reference Sequence NR_025787). It has been proposed that a genotypic difference of >1% is sufficient to be named as a new species [9] and where there are distinct phenotypic differences between the related species, the separation as a new species is even more strongly warranted. BLAST sequence analysis showed that NTM165* hsp65* had a 99% (411/414 nt) DNA identity to* M. cosmeticum* (GenBank accession number AY449731.1) whilst the closest match to the* rpoB* sequence was* M. canariasense* (GenBank accession number KJ720535.1) (677/696 nt, 97%). The ITS sequence was also searched against the GenBank database and the closest result was a 94% identity to* M. neoaurum* (GenBank accession number CP006936.2). The absence of* M. cosmeticum* and* M. canariasense* ITS sequences in the GenBank database likely accounted for* M. neoaurum* as the best match.

Biochemical characterization of NTM165 was performed as described by Metchock et al. [10]. The isolate grew rapidly on MacConkey agar and on 5% NaCl Lowenstein-Jensen medium (2 to 3 days). It was negative for nitrate reductase, arylsulfatase, and Tween 80 hydrolysis (Table 1). Therefore, NTM165 was judged to be biochemically and phenotypically distinct from related species based on morphology, single carbon source utilization, and nitrate reduction tests (Table 1). Susceptibility testing was performed and interpreted according to Clinical and Laboratory Standards Institute (CLSI) guidelines [11]. The isolate was susceptible to amikacin, cefoxitin, ciprofloxacin, moxifloxacin, clarithromycin (up to 14 days of incubation), doxycycline, imipenem, linezolid, and sulfamethoxazole. The susceptibility profile of NTM165 was similar to related species of* M. cosmeticum* and* M. canariasense*, which were also highly susceptible to antimycobacterial drugs [12, 13].

## 3. Discussion

MALDI-ToF provided the first clues that this organism was highly similar to* M. cosmeticum*. However, subsequent molecular analysis showed that NTM165 was genetically dissimilar to the closest matches of* M. cosmeticum* and* M. canariasense*, having <100% identity for the 16S rRNA,* hsp65*, and* rpoB* gene sequences. Additionally, comparative biochemical assays and growth features showed NTM165 was uniquely different to another related* Mycobacterium*. For the identification of* M. canariasense*, there were reported difficulties with its accurate identification using HPLC, matching it to* M. fortuitum* [14]. Our experience with HPLC identification also parallels previous reports where it has been shown that the technique is inadequate in differentiating species with shared patterns, particularly amongst the fast growers [12–14].

The first reported strains of* M. cosmeticum* were obtained from cultures of a sink drain in a nail salon and from a granulomatous lesion of a female mesotherapy patient in Venezuela [15]. The pathogenicity of* M. cosmeticum* was further demonstrated in additional cases whereby the bacterium was responsible for pulmonary disease, catheter-associated bacteremia and most recently as a colitogenic agent [16, 17].* M. canariasense*, akin to* M. cosmeticum*, is an opportunistic pathogen linked to bacteremia in patients with underlying malignant diseases [12, 13, 18]. Therefore, it is not inconceivable that NTM165, being related to these two species, has also pathogenic potential.

## 4. Conclusion

In this report, we describe for the first time the isolation from an ear swab sample of a patient with chronic otitis externa, a novel* M. cosmeticum*-like NTM, which is believed to be the etiologic agent of the infection. In this study, we highlight the importance of the use of a multiple-pronged approach for the identification and characterization of the microorganism, in particular, the importance of sequencing housekeeping genes to further differentiate closely related mycobacterial species.

The 16S rRNA,* hsp65, rpoB*, and ITS sequences of NTM165 have been deposited in GenBank under the respective accession numbers of KP012254, KP012255, KP012256, and KP012257.

## Figures and Tables

**Table 1:** Phenotypic characteristics of NTM165 and closely related rapidly growing mycobacteria.

	NTM165	*M. cosmeticum* ^†^	*M. canariasense* ^†^	*M. brisbanense* ^†^ (*M. fortuitum* complex)	*M. neoaurum* ^†^
Colony morphology	Off-white, flat, and irregular margin. Initially smooth becoming wrinkled with age. Nonpigmented	Smooth, dome-shaped, and opaque. Pigmented	Pale yellow, smooth, moist, and shiny. Nonpigmented	Off-white, mucoid, convex, round, and entire-edged. Nonpigmented	Smooth, yellow. Pigmented
Growth on MacConkey agar	+	+	+	+	−
Growth on 5% NaCl	+	−	−	+	+
Nitrate reductase	−	+	−	+	V
Tween hydrolysis	−	NA	+	NA	V
Arylsulfatase (3 days)	−	−	+	+	−
Utilization of the following:					
Citrate	−	+	−	−	−
D-Mannitol	−	+	+	+	+
*myo*-Inositol	−	+	+	+	+
Sorbitol	−	−	NA	+	−
Acid production from the following:					
Arabinose	+	+	+	+	V
Xylose	+	+	+	+	V
Dulcitol	−	NA	−	−	−

^†^Phenotypic characteristics of the *M.  cosmeticum*, *M.  canariasense*, *M.  brisbanense,* and *M.  neoaurum *isolates were obtained from their respective publications [12, 14, 15, 19].

V: variable.

NA: data not available.

## References

[1] van Ingen J., Looijmans F., Mirck P., Dekhuijzen R., Boeree M., van Soolingen D. (2010). Otomastoiditis caused by *Mycobacterium abscessus*, The Netherlands. *Emerging Infectious Diseases*.

[2] Linmans J. J., Stokroos R. J., Linssen C. F. M. (2008). Mycobacterium abscessus, an uncommon cause of chronic otitis media: a case report and literature review. *Archives of Otolaryngology—Head and Neck Surgery*.

[3] Muller B., Kemper J., Hartwig N. G., Mooi-Kokenberg E. A. N. M., van Altena R., Versteegh F. G. A. (2006). *Mycobacterium avium* intracellulare otomastoiditis: case report and literature review. *European Journal of Clinical Microbiology and Infectious Diseases*.

[4] Plemmons R. M., McAllister C. K., Liening D. A., Garces M. C. (1996). Otitis media and mastoiditis due to *Mycobacterium fortuitum*: case report, review of four cases, and a cautionary note. *Clinical Infectious Diseases*.

[5] Clark W. B., Brook I., Bianki D., Thompson D. H. (1997). Microbiology of otitis externa. *Otolaryngology: Head and Neck Surgery*.

[6] Fusconi M., Petrozza V., Taddei A. R. (2011). Is biofilm the cause of chronic otitis externa?. *Laryngoscope*.

[7] Weisburg W. G., Barns S. M., Pelletier D. A., Lane D. J. (1991). 16S ribosomal DNA amplification for phylogenetic study. *Journal of Bacteriology*.

[8] Ngan G. J. Y., Ng L. M., Jureen R., Lin R. T. P., Teo J. W. P. (2011). Development of multiplex PCR assays based on the 16S-23S rRNA internal transcribed spacer for the detection of clinically relevant nontuberculous mycobacteria. *Letters in Applied Microbiology*.

[9] Tortoli E. (2003). Impact of genotypic studies on mycobacterial taxonomy: the new mycobacteria of the 1990s. *Clinical Microbiology Reviews*.

[10] Metchock B. G., Nolte F. S., Wallace R. J., Murray P. R., Baron E. J., Pfaller M. A., Tenover F. C., Yolken R. H. (1999). Mycobacterium. *Manual of Clinical Microbiology*.

[11] Clinical and Laboratory Standards Institute (2011). *Susceptibility Testing of Mycobacteria, Nocardia, and Other Aerobic Actinomycetes*.

[12] Soledad Jiménez M., Campos-Herrero M. I., García D., Luquin M., Herrera L., García M. J. (2004). *Mycobacterium canariasense* sp. nov. *International Journal of Systematic and Evolutionary Microbiology*.

[13] Paniz-Mondolfi A., Ladutko L., Brown-Elliott B. A. (2014). First report of *Mycobacterium canariasense* catheter-related bacteremia in the Americas. *Journal of Clinical Microbiology*.

[14] Schinsky M. F., Morey R. E., Steigerwalt A. G. (2004). Taxonomic variation in the Mycobacterium fortuitum third biovariant complex: description of *Myobacterium boenickei* sp. nov., *Mycobacterium houstonense* sp. nov., *Mycobacterium neworleansens*e sp. nov. and *Mycobacterium brisbanense* sp. nov. and recognition of *Mycobacterium porcinum* from human clinical isolates. *International Journal of Systematic and Evolutionary Microbiology*.

[15] Cooksey R. C., de Waard J. H., Yakrus M. A. (2004). *Mycobacterium cosmeticum* sp. nov., a novel rapidly growing species isolated from a cosmetic infection and from a nail salon. *International Journal of Systematic and Evolutionary Microbiology*.

[16] Cooksey R. C., De Waard J. H., Yakrus M. A. (2007). *Mycobacterium cosmeticum*, Ohio and Venezuela. *Emerging Infectious Diseases*.

[17] Boschetti G., Cotte E., Moussata D. (2011). Identification of *Mycobacterium cosmeticum* sp. as a novel colitogenic infectious agent in a nonimmunocompromised patient. *Inflammatory Bowel Diseases*.

[18] Campos-Herrero M. I., García D., Figuerola A., Suárez P., Campo C., García M. J. (2006). Bacteremia caused by the novel species *Mycobacterium canariasense*. *European Journal of Clinical Microbiology and Infectious Diseases*.

[19] Davison M. B., McCormack J. G., Blacklock Z. M., Dawson D. J., Tilse M. H., Crimmins F. B. (1988). Bacteremia caused by *Mycobacterium neoaurum*. *Journal of Clinical Microbiology*.

